# Prediction of TERTp-mutation status in IDH-wildtype high-grade gliomas using pre-treatment dynamic [^18^F]FET PET radiomics

**DOI:** 10.1007/s00259-021-05526-6

**Published:** 2021-09-07

**Authors:** Zhicong Li, Lena Kaiser, Adrien Holzgreve, Viktoria C. Ruf, Bogdana Suchorska, Vera Wenter, Stefanie Quach, Jochen Herms, Peter Bartenstein, Jörg-Christian Tonn, Marcus Unterrainer, Nathalie L. Albert

**Affiliations:** 1 Department of Nuclear Medicine, University Hospital, LMU Munich, Marchioninistr. 15, 81377 Munich, Germany; 2 Center for Neuropathology and Prion Research, LMU Munich, Munich, Germany; 3 Department of Neurosurgery, University Hospital, LMU Munich, Munich, Germany; 4 Department of Neurosurgery, Sana Hospital, Duisburg, Germany; 5 German Cancer Consortium (DKTK), Partner Site Munich, German Cancer Research Center (DKFZ), Heidelberg, Germany; 6 Department of Radiology, University Hospital, LMU Munich, Munich, Germany

**Keywords:** Radiomics, [^18^F]FET PET, TERTp-mutation, Glioma

## Abstract

**Purpose:**

To evaluate radiomic features extracted from standard static images (20–40 min p.i.), early summation images (5–15 min p.i.), and dynamic [^18^F]FET PET images for the prediction of TERTp-mutation status in patients with IDH-wildtype high-grade glioma.

**Methods:**

A total of 159 patients (median age 60.2 years, range 19–82 years) with newly diagnosed IDH-wildtype diffuse astrocytic glioma (WHO grade III or IV) and dynamic [^18^F]FET PET prior to surgical intervention were enrolled and divided into a training (*n* = 112) and a testing cohort (*n* = 47) randomly. First-order, shape, and texture radiomic features were extracted from standard static (20–40 min summation images; TBR_20–40_), early static (5–15 min summation images; TBR_5–15_), and dynamic (time-to-peak; TTP) images, respectively. Recursive feature elimination was used for feature selection by 10-fold cross-validation in the training cohort after normalization, and logistic regression models were generated using the radiomic features extracted from each image to differentiate TERTp-mutation status. The areas under the ROC curve (AUC), accuracy, sensitivity, specificity, and positive and negative predictive value were calculated to illustrate diagnostic power in both the training and testing cohort.

**Results:**

The TTP model comprised nine selected features and achieved highest predictability of TERTp-mutation with an AUC of 0.82 (95% confidence interval 0.71–0.92) and sensitivity of 92.1% in the independent testing cohort. Weak predictive capability was obtained in the TBR_5–15_ model, with an AUC of 0.61 (95% CI 0.42–0.80) in the testing cohort, while no predictive power was observed in the TBR_20–40_ model.

**Conclusions:**

Radiomics based on TTP images extracted from *dynamic* [^18^F]FET PET can predict the TERTp-mutation status of IDH-wildtype diffuse astrocytic high-grade gliomas with high accuracy preoperatively.

**Supplementary Information:**

The online version contains supplementary material available at 10.1007/s00259-021-05526-6.

## Introduction

Mutations in the telomerase reverse transcriptase promoter (TERTp), leading to telomerase activation and lengthened telomeres, play an important role in the formation of brain cancer and individual prognosis [1–3]. In diffuse astrocytic high-grade gliomas without mutation of the isocitrate dehydrogenase gene (IDH-wildtype), TERTp mutations are reported to be associated with poor overall survival [4–6]. Molecular genetic analysis of the TERTp-mutation status has therefore gained increasing attention in the clinical routine diagnosis of IDH-wildtype diffuse astrocytic gliomas and will be included in the upcoming glioma WHO classification [7–9].

Molecular imaging using positron emission tomography (PET) with radiolabelled amino acids such as *O*-(2-[^18^F]-fluoroethyl)-L-tyrosine ([^18^F]FET) is a useful tool for the characterization and evaluation of primary brain neoplasms [10–12], and its application in the clinical management of brain tumour patients has been recommended by the Response Assessment in Neuro-Oncology (RANO) Working Group [13–17]. While static image data (standard 20–40 min summation images) are particularly used for the delineation of the tumour extent, the assessment of *dynamic* [^18^F]FET PET data has been shown to provide additional information about tumour biology [18]. More aggressive gliomas (i.e. high-grade gliomas and/or IDH-wildtype gliomas) were shown to be characterized by a high tracer uptake within the first 5–15 min post injection (p.i.) with subsequent curve decrease, while less aggressive gliomas (i.e. low grade gliomas and/or IDH-mutant gliomas) typically show a slowly increasing [^18^F]FET uptake with highest values in the later time frames [12, 19, 20]. As the early peak uptake in aggressive gliomas is missed in the standard 20–40 min p.i. summation images, it does not surprise that the maximal tumour-to-background ratio (TBR_max_) evaluation obtained in early summation images (5–15 min p.i.) was reported to perform better than the standard static TBR_max_ values (20–40 min p.i.) for the differentiation between low-grade and high-grade gliomas [17], which led to the suggestion to include these early summation images for a better glioma characterization. Another interesting parameter derived from dynamic [^18^F]FET PET is the minimal time-to-peak (TTP_min_), which is extracted from the time-activity-curves and was reported to provide prognostic information [21]. Interestingly, an early TTP_min_ was associated with an aggressive disease course in newly diagnosed gliomas and was able to predict an IDH-wildtype status [22, 23]. Yet, in our recently published study investigating [^18^F]FET uptake characteristics in TERTp mutant and TERTp wildtype glioblastomas, neither the standard TBR_max_ as static parameter nor TTP_min_ as dynamic parameter were associated with the TERTp-mutation status [24].

In recent years, radiomics have been increasingly investigated as a promising non-invasive tool for accurate diagnosis and prognosis assessment by converting medical images into high-dimensional quantitative image features and establishing predictive models [25–32]. However, radiomics have not been applied for the detection of TERTp mutations on [^18^F]FET PET images so far. Therefore, the aim of this study was to evaluate radiomic features extracted from standard static images (20–40 min p.i.), early summation images (5–15 min p.i.) as well as dynamic [^18^F]FET PET images for the prediction of the TERTp-mutation status in patients with newly diagnosed IDH-wildtype diffuse astrocytic high-grade glioma.

## Materials and methods

### Patients

Patients with primary diagnosis of a glioma who had received a pre-treatment dynamic [^18^F]FET PET scan at the Department of Nuclear Medicine of the LMU Munich between December 2005 and June 2016 were screened for this retrospective study. Inclusion criteria were (1) neuropathologically confirmed IDH-wildtype diffuse astrocytic gliomas (WHO grade III or IV) according to the updated 2016 WHO classification [33], (2) availability of the TERTp-mutation status, and (3) pre-treatment dynamic [^18^F]FET PET scan (ECAT EXACT HR + , Siemens Healthineers, Inc., Erlangen, Germany Siemens Medical Systems, Inc., Erlangen, Germany). [^18^F]FET-negative gliomas (tumour-to-background ratio, TBR < 1.6) were excluded. All patients had given written informed consent prior to the PET scan as part of the clinical routine. The retrospective analysis of PET imaging data was approved by the institutional ethics committee (604–16). A total of 61% of the investigated patients (97/159) have been evaluated in a previous study [24].

### Histopathology and molecular genetic analysis

Histopathology and molecular genetic analyses were performed at the Institute of Neuropathology, LMU Munich, Germany. All patients initially classified according to the 2007 WHO brain tumour classification [34] were re-classified according to the 2016 WHO classification [33]. The IDH-mutation status and TERTp-mutation status were evaluated according to clinical standard protocols [35, 36].

### [^18^F]FET PET imaging

[^18^F]FET PET scans were performed at the Department of Nuclear Medicine, LMU Munich, Germany. Images were acquired by using an ECAT EXACT HR + PET scanner (Siemens Healthineers, Inc., Erlangen, Germany) with the standard protocol [11, 37]. Exactly 180 MBq of [^18^F]FET were injected after a 15-min transmission scan with a ^68^Ge rotating rod source. After tracer injection up to 40 min post injection in 3-D mode consisting of 16 frames (7 × 10 s, 3 × 30 s, 1 × 2 min, 3 × 5 min, and 2 × 10 min) with a reconstructed voxel size of 2.03 × 2.03 × 2.43 mm^3^ and matrix size of 128 × 128 × 63, dynamic emission recording was finished. Two-dimensional filtered back-projection reconstruction algorithm using a 4.9-mm Hann Filter was applied for image reconstruction, then corrected for attenuation, decay, dead time, and random and scattered coincidences. When relevant motion was visible in dynamic PET data, a frame-wise correction was performed by using PMOD fusion tool (version 3.5, PMOD Technologies, Zurich, Switzerland) after frame-wise checking for motion.

### Segmentation of tumour volumes and brain background

First, a background activity was extracted from a large crescent-shaped volume of interest (VOI) in the contralateral healthy hemisphere as published previously [38]. For tumour segmentation, a VOI was drawn using a TBR-threshold of 1.6 in static 20–40 min p.i. summation images as suggested by Pauleit et al. [39]. All segmentations were processed within the PMOD View tool (version 3.5, PMOD Technologies, Zurich, Switzerland).

### Image normalization and TTP image generation

We used the in-house developed software described previously by Kaiser et al. [40] (C +  + with integration of the ROOT data analysis framework, version 6.22/08, Cern, Switzerland and ITK segmentation and registration toolkit 4.13.3, National Library of Medicine) to generate voxel-wise parametric images. Then we normalized the image values with the mean background value derived from each image by using the VOI of background to generate early 5–15 min p.i. (TBR_5–15_) and late 20–40 min p.i. (TBR_20–40_) TBR images. For TTP images, time-activity curves (TAC) were extracted from each voxel, which were then classified according to the time frame reaching the peak uptake (i.e. (1) < 5 min, (2) 5–10 min, (3) 10–15 min, (4) 15–20 min, (5) 20–30 min, and (6) 30–40 min). To avoid influence from early blood flush, TTP analyses did not include the first 2.7 min p.i. [40]. In case of a positive late slope (15–40 min p.i.), the TTP was always assigned to group 6.

### Radiomic feature extraction

Radiomic features from parametric images were extracted with PyRadiomics (version 3.0.1) [41] as introduced previously by Kaiser et al. [42], and complied with the Imaging Biomarker Standardization Initiative (IBSI) guidelines [43]. Before extraction, images were resampled to isotropic voxels using linear interpolation in PyRadiomics (size 2.03 × 2.03 × 2.03 mm ^3^). Classes of features extracted from TBR_5–15_, TBR_20–40_, and TTP images included first-order features, shape features, and texture features. No image filters were used. The chosen fixed intensity bin size was set to the average interquartile range divided by 4, which led to 0.18 for TBR_5–15_ images and 0.13 for TBR_20–40_ images [42, 44]. As the smallest time frame duration considered in the TTP categories was 5 min, this was used as the fixed bin width for radiomics calculation of TTP images.

### Feature selection

Before feature extraction, a stratified random split was used to assign 70% of the patients to the training cohort (*n* = 112) and the remaining 30% to the testing cohort (*n* = 47), with a balanced distribution of TERTp-wildtype and TERTp-mutation.

Features were standardized as follows: for each feature, we calculated the mean value and the standard deviation. The mean value was subtracted from each individual value, which was then divided by the standard deviation. Feature normalization was computed only in the training cohort and then applied on the testing cohort. Since the number of features was large, we compared the similarity of each feature pair. If the Pearson correlation coefficient (PCC) value of the feature pair was larger than 0.99, we removed one of them. After this process, the number of the features was reduced and each feature was independent to each other. The recursive feature elimination (RFE) based on logistic regression classifier was performed to reduce redundant features and select potential TERTp-mutation related features [45]. Considering the imbalance of comparison groups, we performed the weighted logistic regression in the ‘balanced’ mode, which gives higher weight to the minority class and lower weight to the majority class and therefore automatically adjusts weights inversely proportional to class frequencies in the input data [46]. Each iteration removes a feature which is considered least important. After stratified split-based 10-fold cross-validation, the area under the receiver operating characteristic curve (AUC) of the model in the training cohort was used to determine the optimal number of features.

### Model construction and testing

Logistic regression (LR) models were built to predict the TERTp-mutation status by fitting the selected radiomic features. Each model was generated by using only the radiomic features extracted from each image (i.e. TBR_5–15_, TBR_20–40_, and TTP images) separately. According to the coefficients of selected features generated by the LR models [47], the risk probability of TERTp-mutation was calculated by the following formula:

$${\rm P}\left(y=1|x;\theta \right)= \frac{1}{1+{e}^{-{\theta }^{\mathrm{\rm T}}x}}$$

$x$ is the value of selected features, $\theta$ is the coefficient of selected features, and ${\theta }_{0}$ represents the intercept. In case of ${\rm P}>0.5$, TERTp-mutation status was considered as positive by the LR model.

**Fig. 1:**
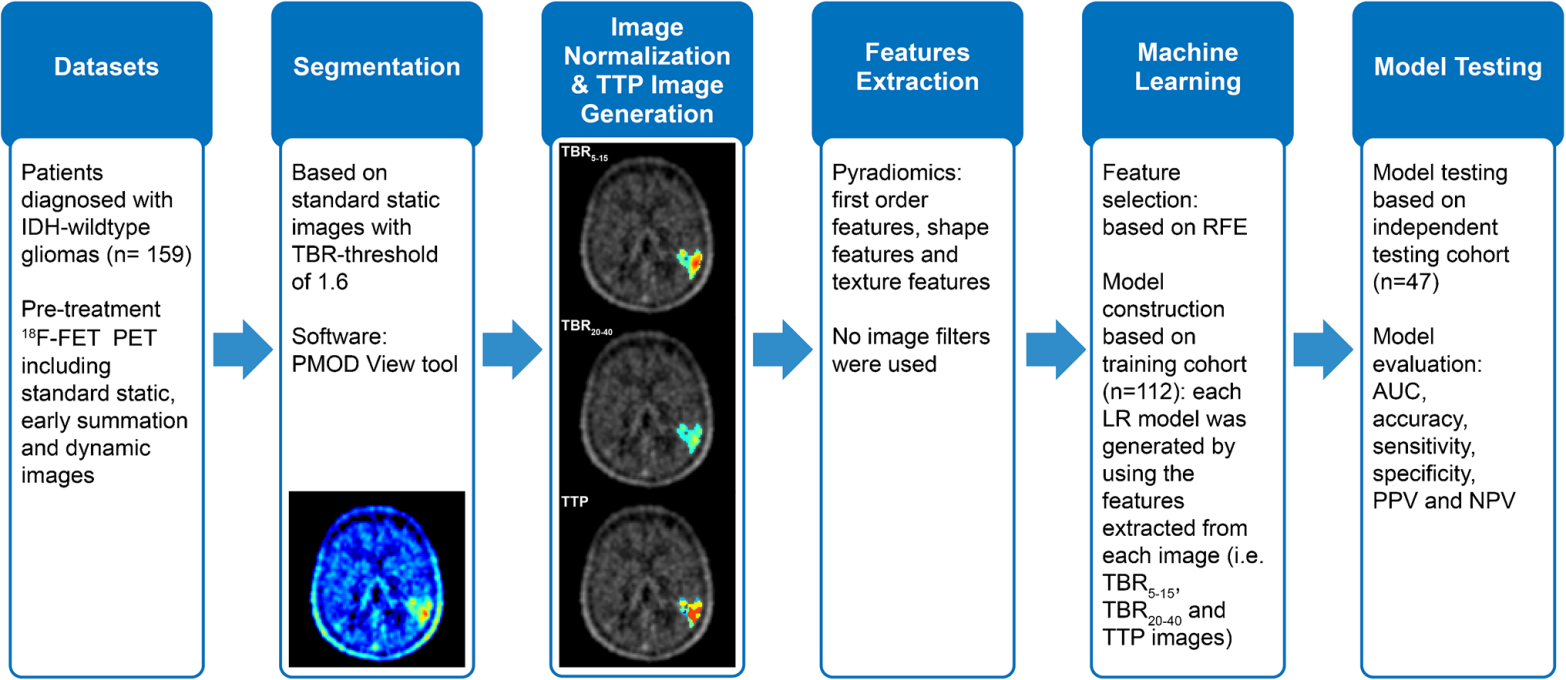
The workflow of process. TBR tumour-to-background ratio, TTP time-to-peak, RFE recursive feature elimination, LR logistic regression, AUC area under the receiver operating characteristic curve, PPV positive predictive value, NPV negative predictive value

Model testing was applied to the independent testing cohort, which was not involved in the process of model training. The workflow of the process is presented in Fig. 1.

### Statistical analysis

To evaluate the model performance, receiver operating characteristic curve (ROC) analysis was performed in the training and testing cohort. The AUC was calculated as quantitative measure to illustrate diagnostic power. The accuracy, sensitivity, specificity, positive predictive value (PPV), and negative predictive value (NPV) were calculated. 95% confidence intervals (CI) were calculated by using a non-parametric bootstrap method, which was repeated 1000 times to get a bootstrap distribution of the results.

Categorical variables or continuous variables were reported as numbers and percentages or as mean and standard deviation. Categorical variables were compared by the *χ*^2^ test, and continuous variables were compared by the Mann–Whitney *U* test. *P* < 0.05 were considered statistically significant. Statistical analyses were programmed in Python (v. 3.8.5; https://www.python.org/).

## Results

### Patient characteristics

A total of 159 patients (median age, 60.2 years; range, 19–82 years) were enrolled in this study. Exactly 31 patients (19.50%) were diagnosed with TERTp-wildtype, and 128 patients had TERTp mutation. The clinical characteristics are presented in Table 1. There were no significant differences between the training and testing cohorts with regard to age, sex, WHO grade, and TERTp mutation status, with TERTp-wildtype rates of 19.64% and 19.15%, respectively.

**Table 1:** Clinical characteristics of the patients

	Training cohort (*n* = 112)		Testing cohort (*n* = 47)		
	TERTp-mutation	TERTp-wildtype	TERTp-mutation	TERTp-wildtype	*P*
Characteristic	(*n* = 90)	(*n* = 22)	(*n* = 38)	(*n* = 9)	0.8958
Age, years	58.1 ± 12.3		59.2 ± 11.2		0.3699
Sex					
Female	45 (40.2%)		17 (54.8%)		0.1449
Male	67 (59.8%)		14 (45.2%)		
WHO grade					
III	39 (34.8%)		14 (29.8%)		0.5389
IV	73 (65.2%)		33 (70.2%)		

Data are means ± standard deviations or numbers of patients with percentages in parentheses. *P* value was derived from the univariate association analyses between each clinical parameter. Calculated by using the independent sample *t* test for continuous variables and the *χ*^2^ test for categoric variables

### Radiomic feature extraction and selection

In this study, 107 radiomic features of candidates were generated from standard static images (20–40 min p.i.), early summation images (5–15 min p.i.), and dynamic [^18^F]FET PET images respectively, including first-order statistics, shape-based features, and texture features. After PCC process, 80 TBR_20–40_ features, 83 TBR_5–15_ features, and 91 TTP features were retained. For the TBR_20–40_ model, based on the AUC of the 10-fold cross-validation on the training cohort, 14 features were finally selected to fit the LR model after performing the RFE method. For the TBR_5–15_ model and the TTP model, 9 features and 10 features were selected for inclusion in the LR model, respectively (Fig. 2).

**Fig. 2:**
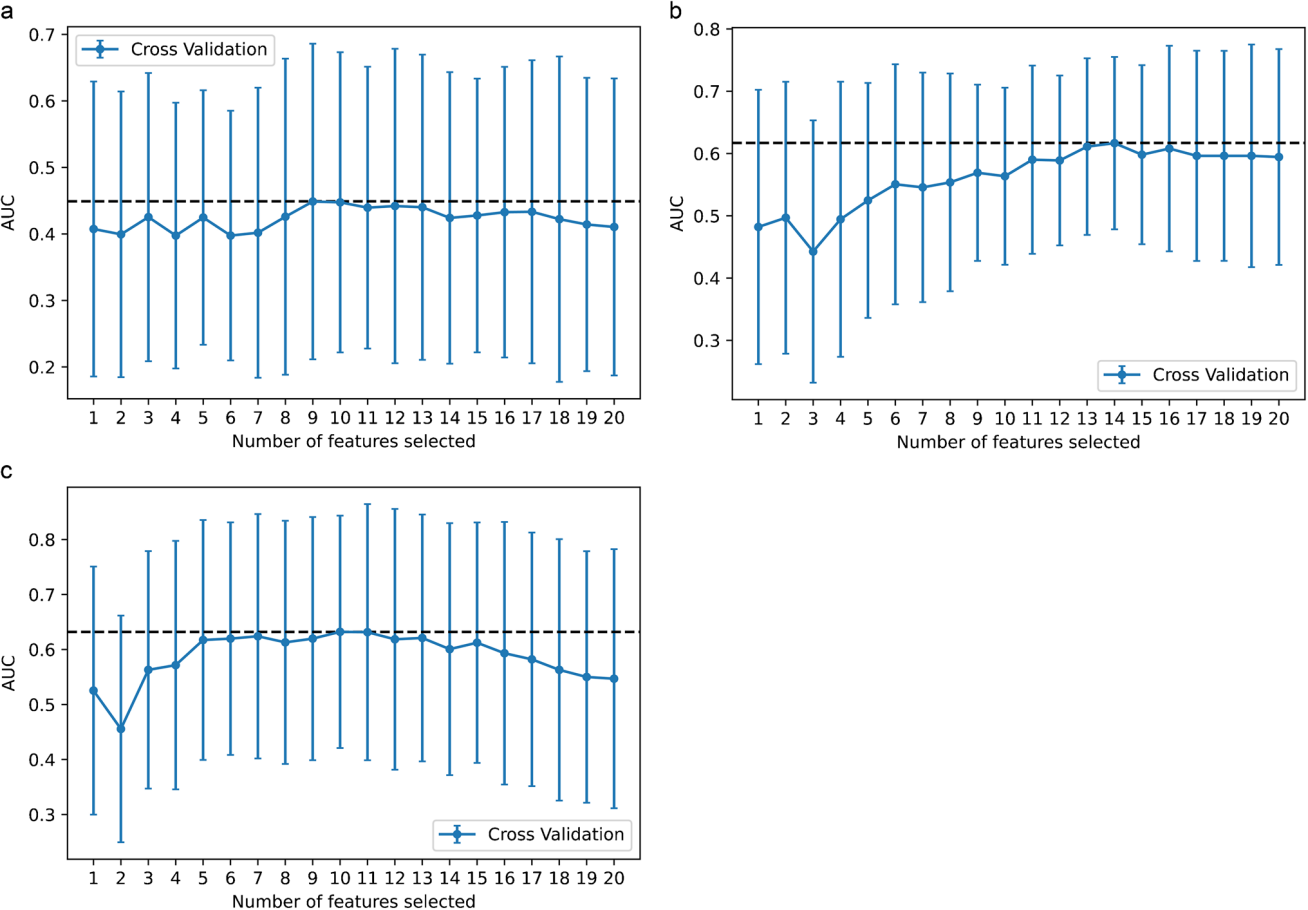
The feature selection process of the RFE method. Each iteration removes a feature that is considered least important and corresponds to a 10-fold cross-validation. After 10-fold cross-validation, the AUC of the model in the training cohort was used to determine the optimal number of features. The minimum AUC of feature number was selected. **a** TBR_5–15_ model, **b** TBR_20–40_, and **c** TTP model; 9, 14, and 10 features were selected respectively. RFE recursive feature elimination, AUC area under the receiver operating characteristic curve

### Diagnostic Validation of the TBR_20–40_ model, TBR_5–15_ model, and TTP model

According to the above-mentioned formula, the risk probabilities of TERTp-mutation were calculated. The coefficients of selected features in the TBR_20–40_ model and TBR_5–15_ model are shown in Table S1. The coefficients of selected features in the TTP model are shown in Table 2.

**Table 2:** Coefficients of selected features in the TTP model

Features	Coefficients
SmallDependenceLowGreyLevelEmphasis	1.508
Energy	1.404
SmallDependenceHighGreyLevelEmphasis	− 1.283
GreyLevelNonUniformityNormalized	− 1.235
LeastAxisLength	− 1.219
Busyness	− 0.916
ShortRunHighGreyLevelEmphasis	− 0.699
Maximum2DDiameterColumn	0.654
LowGreyLevelZoneEmphasis	− 0.626
LargeDependenceHighGreyLevelEmphasis	0.606

Intercept ${\theta }_{0}$ is 0.599 in the TTP model. Details of features were shown in Supplementary Information

**Fig. 3:**
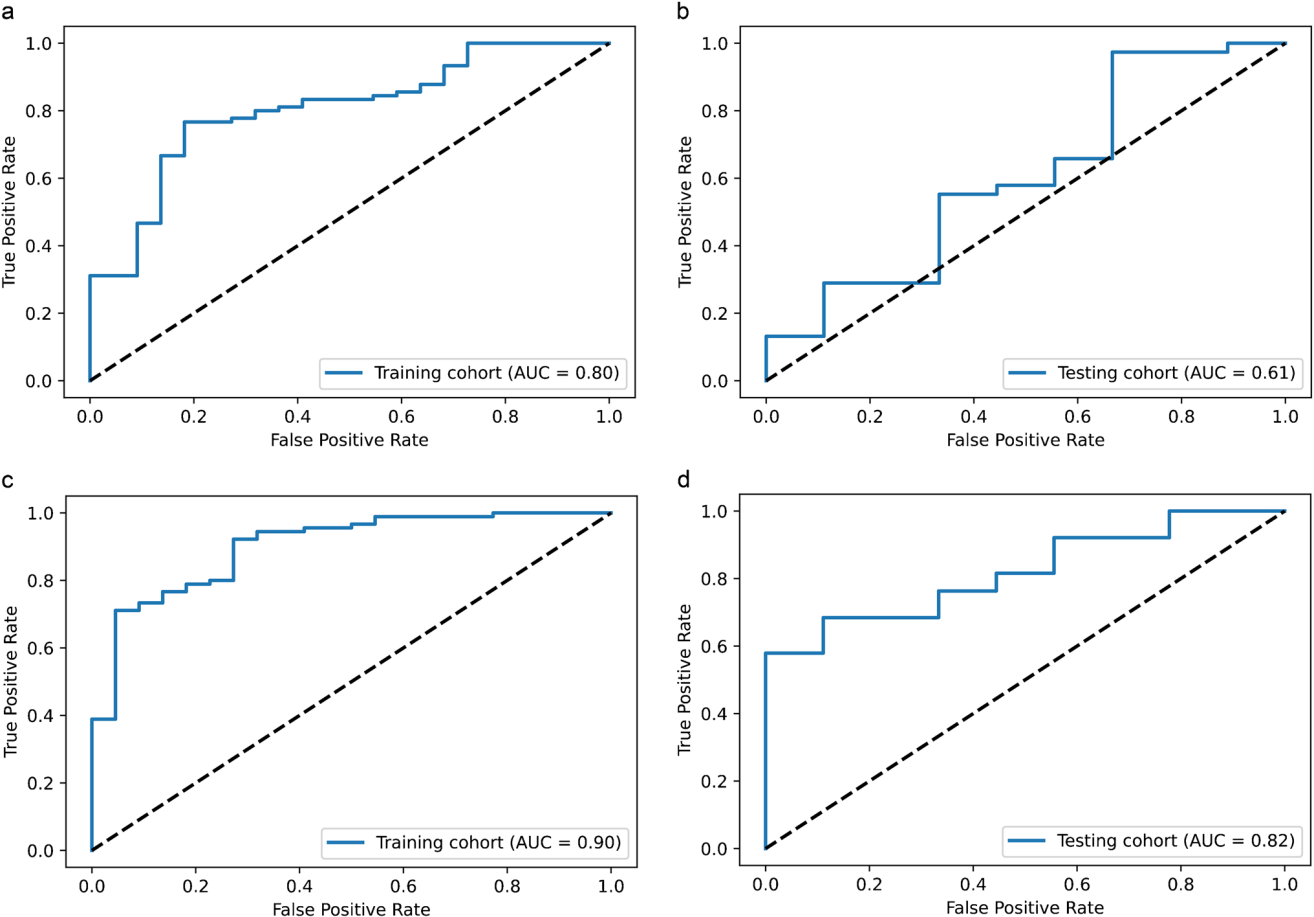
**a** TBR_5–15_ model reached an AUC of 0.80 in the training cohort, and **b** an AUC of 0.61 in the testing cohort. **c** TTP model reached an AUC of 0.90 in the training cohort, and **d** an AUC of 0.82 in the testing cohort. AUC area under the receiver operating characteristic curve

No predictive power was observed in the TBR_20–40_ model with an AUC of only 0.49 (95% CI 0.30–0.69) in the testing cohort (AUC of 0.90 in the training cohort (95% CI 0.85–0.95); see Fig. S1). The TBR_5–15_ model demonstrated weak predictive capability to predict a TERTp-mutation (Fig. 3a, b), with an AUC of 0.61 (95% CI 0.42–0.80) in the testing cohort and an AUC of 0.80 (95% CI 0.71–0.89) in the training cohort. The TTP model showed the strongest predictive power and achieved an AUC of 0.82 (95% CI 0.71–0.92) and 0.90 (95% CI 0.84–0.95) in the testing cohort and training cohort, respectively (Fig. 3c, d).

Detailed information about the performance of each model is shown in Table 3.

**Table 3:** Performance of each model

	TBR_5–15_		TBR_20–40_		TTP	
	Training cohort	Testing cohort	Training cohort	Testing cohort	Training cohort	Testing cohort
AUC	0.80	0.61	0.90	0.49	0.90	0.82
AUC 95%CI	(0.71–0.89)	(0.42–0.80)	(0.85–0.95)	(0.30–0.69)	(0.84–0.95)	(0.71–0.92)
Accuracy	0.75.0%	66.0%	83.0%	66.0%	78.6%	83.0%
Sensitivity	73.3%	73.7%	81.1%	73.7%	77.8%	92.1%
Specificity	81.8%	33.3%	90.9%	33.3%	81.8%	44.4%
PPV	94.3%	82.4%	97.3%	82.4%	94.6%	87.5%
NPV	42.9%	23.1%	54.1%	23.1%	47.4%	57.1%

*CI* confidence interval

## Discussion

Our study showed that radiomics based on *dynamic* [^18^F]FET PET data can reliably predict the TERTp-mutation status of IDH-wildtype diffuse astrocytic high-grade gliomas. Best predictability was reached using the TTP model derived from dynamic PET, and weak predictive capability was obtained with radiomics based on early summation images (5–15 min p.i.), while no reliable information about the TERTp-mutation status was possible based on the standard summation images (20–40 min p.i.).

Previous studies have shown that patients with IDH-wildtype TERTp-mutant glioblastoma have a significantly shorter progression free and overall survival compared to those with TERT-wildtype status. Therefore, TERTp-mutation status is now considered to be an important diagnostic and prognostic factor in primary glioblastomas and especially in patients with IDH-wildtype glioma [3, 5, 8, 9, 48]. TERTp-mutations indicate tumours that require aggressive and immediate treatments [3]. Hence, a preoperative tool for the prediction of a TERTp-mutation would be useful for early decision making and clinical management of patients with suspected glioma.

Several studies have analyzed the value of MRI based radiomics to predict the TERTp-mutation status in brain tumour patients [49–51]. Although these studies reported to achieve high accuracy values in the range of 79.88–93.80%, only WHO grade II or/and III gliomas have been considered and a limited number of patients has been investigated [49–51]. Besides, Tian et al. established a multiparameter MRI based radiomics model for the prediction of the TERTp-mutation status in patients with high-grade glioma [52], but ignored that TERTp-mutations play different roles in different IDH phenotypes [48].

Compared with conventional MRI, amino acid PET has been shown to be more sensitive in the definition of brain tumour extent [39], and dynamic [^18^F]FET uptake parameters extracted from the TAC have shown to be an independent biomarker for prognosis [53, 54]. Several studies have reported the informative value of [^18^F]FET PET-based radiomics in personalized clinical decisions and individualized treatment selection [27–29, 55]. Lohmann et al. found textural feature analysis in combination with TBRs to better differentiate brain metastasis recurrence from radiation injury than TBRs alone, and [^18^F]FET PET radiomics achieved a higher accuracy than the best standard FET PET parameter (TBR_max_) to diagnose patients with pseudoprogression [27, 55]. Haubold et al. utilized multiparametric [^18^F]FET PET/MRI and MR fingerprinting to decode and phenotype cerebral gliomas, which may serve as an alternative to invasive tissue characterization [28]. In addition, Carles et al. evaluated the prognostic value of [^18^F]FET PET radiomics after re-irradiation, and found it could contribute to the selection of recurrent glioblastoma patients benefiting from re-irradiation [29]. However, all studies included radiomics based on standard *static* images (20–40 min p.i.) only and did not extract radiomic features derived from *dynamic* [^18^F]FET PET as well as early summation images (5–15 min p.i.) even though two studies have shown the impact of *dynamic* parameters on radiomics [32, 56]. Furthermore, no study has evaluated the potential to predict the TERTp-mutation status by [^18^F]FET PET radiomics so far.

This study included standard static images (20–40 min p.i.), early summation images (5–15 min p.i.), and *dynamic* [^18^F]FET PET images to develop the radiomic models. A total of 107 features were extracted from each image. Our TTP model, built from ten dynamic [^18^F]FET PET features selected by RFE, achieved the highest AUC of 0.82 in the independent testing cohort, indicating that the TERTp-mutation status can be predicted by using [^18^F]FET PET based radiomics. Notably, our former study did neither find an association between the TERTp-mutation status and traditional static [^18^F]FET PET parameters (TBR_max_ and TBR_mean_ in static 20–40 min summation images) nor the standard dynamic parameter TTP_min_ [24].

Interestingly, radiomics based on the standard TBR_20–40_ model showed a low performance for the prediction of the TERTp-mutation status, and even the TBR_5–15_ model, generated from nine early summation [^18^F]FET PET features, had an accuracy of only 66% and an AUC of 0.61 in the testing cohort. With a high prediction accuracy of 83% in the TTP model, our study demonstrates that radiomic features extracted from *dynamic* PET data can achieve a higher performance level than models based on static PET data. Remarkably, the sensitivity of the TTP model reached 92.1% in the testing cohort, so that patients with aggressive TERTp-mutant glioma can be identified non-invasively with high probability [3]. With the generated multivariate LR-based formula, health practitioners will be able to calculate the patient individual risk probability of bearing a TERTp-mutation before neurosurgical intervention. Our study shows that even sophisticated radiomic analysis of static [^18^F]FET PET imaging cannot replace dynamic acquisitions, at least with regard to the prediction of the TERTp-mutation status.

Traditional dynamic [^18^F]FET PET parameters such as the classification of the time-activity curve (increasing vs. decreasing or increasing vs. plateau vs. decreasing), the slope or the TTP_min_ were most frequently calculated from a mean VOI-TAC of the tumour or from the hot-spot of the tumour with a 90% isocontour [10, 12, 19]. Considering the heterogeneity of gliomas, it may happen that the hot-spot in standard summation images does not correspond to the most suspicious tumour aggressiveness when only considering TTP_min_ and TAC and that, therefore, the most aggressive areas are inadvertently not evaluated. In contrast, we extracted the dynamic [^18^F]FET uptake information in every voxel within the tumour VOI and generated TTP images. This approach, which was first introduced by Kaiser et al. [40, 42], ensures that the dynamic information including the heterogeneity of uptake kinetics is extracted and that radiomics can be performed on the prognostically valuable dynamic data. The correlation between tumour heterogeneity and TERTp-mutation status can be considered in GreyLevelNonUniformityNormalized (GLNN) feature, which was used in the TTP model (see Table 2). GLNN belongs to Gray Level Dependence Matrix (GLDM), which is mathematically equal to first order–uniformity and is a measure of the homogeneity of the image array. A low value implies a greater heterogeneity, which was correlated with the TERTp-mutation, indicating that tumours with more heterogeneous TTP images are more likely to be classified as TERTp-mutant glioma.

Several limitations of this study should be discussed. First, the number of investigated patients is relatively small. However, it needs to be considered that we analyzed a very homogeneous group of patients with newly diagnosed and untreated IDH-wildtype diffuse astrocytic high-grade glioma. To exclude any influence by scanner type, all images in this study were derived from the same PET scanner, which limited the number of patients as well. In order to increase the number of patients, multi-centre validation studies are needed which, however, require phantom studies and harmonization of reconstruction parameters to make images from different PET scanners comparable. Another approach to directly harmonize features extracted from different devices may be to use the ComBat method [57]. In addition, our results are difficult to extrapolate to other centres, as the PET images analyzed in this study were acquired with our old PET scanner with fixed time frames, resulting in relatively long time frames (predominantly 5 and 10 min) in the dynamic analysis which could not be changed afterwards, and were reconstructed using filtered back-projection, while most PET centres now use other reconstruction methods such as ordered subset expectation maximization (OSEM). Furthermore, radiomic features were only extracted from the [^18^F]FET-positive tumour VOI to construct the model. Besides the tumour VOI, the remaining image (with normal seeming tissue) may still contain invisible but useful information. To analyze the entire images, deep learning methods will be necessary. Furthermore, our study focused on PET-based radiomics only. A combination with MRI may improve the performance of the prediction model and should be evaluated in future studies.

## Conclusion

While conventional [^18^F]FET PET parameters assessed by standard analyses have previously shown no association with the TERTp-mutation status, radiomic models can predict the TERTp-mutation status of IDH-wildtype diffuse astrocytic high-grade gliomas with high accuracy preoperatively. Notably, this is only the case for radiomics based on *dynamic* image data (TTP model) instead of standard summation images (20–40 min). Further external validation in multi-centre studies with a larger number of patients is needed to evaluate the potential for clinical applications.

## Supplementary Information

Below is the link to the electronic supplementary material.

Supplementary file1 (DOCX 102 KB)

## Data Availability

The data that support the findings of this study are available on request from the corresponding author, N.L.A. The data are not publicly available due to the privacy of research participants.

## Code availability

The codes that support the findings of this study are available from the Z.L. and L.K., upon reasonable request.
