# Supplementary material for: Prediction of TERTp-mutation status in IDH-wildtype high-grade gliomas using pre-treatment dynamic [18F]FET PET radiomics
Source: Eur J Nucl Med Mol Imaging. 2021 Sep 7;48(13):4415–25. doi: 10.1007/s00259-021-05526-6 (PMC8566644; doi:10.1007/s00259-021-05526-6)
Supplement: Supplementary file 1 — Supplementary file1 (DOCX 102 KB) [file 259_2021_5526_MOESM1_ESM.docx]

**Supplementary Material**

**Radiomics features**

In this study, 107 radiomics features of candidates were generated from standard static images (20-40 min post injection, p.i.), early summation images (5-15 min p.i.) as well as dynamic ^18^F-FET PET images respectively, including first-order statistics, shape-based and texture features. First order features describe the distribution of grey values in VOIs, such as range, entropy and variance. Shape-based features describe the three-dimensional size and shape of the VOIs, such as volume, surface area and maximum diameter. Texture features reflects the texture characteristics of tumor in terms of the interrelationship between pixels, including grey level co-occurrence matrix (GLCM), grey level run length matrix (GLRLM), grey level size-zone matrix (GLSZM), neighborhood grey level different matrix (NGLDM) and grey level dependence matrix (GLDM) features.

The detailed description of each feature can be found in the Pyradiomics documentation(https://pyradiomics.readthedocs.io/en/latest/).

**Supplementary Table**

**Table S1**: Coefficients of selected features in the TBR_5-15_ and TBR_20-40_ model

| TBR_5-15_ | | TBR_20-40_ | |
| --- | --- | --- | --- |
| Features | Coefficients | Features | Coefficients |
| ZoneEntropy | 1.272 | DependenceVariance | -1.279 |
| Complexity | 1.129 | LeastAxisLength | -1.189 |
| ZonePercentage | -1.100 | Maximum2DDiameterColumn | 1.049 |
| Maximum2DDiameterColumn | 0.903 | ClusterProminence | 1.036 |
| LeastAxisLength | -0.836 | Correlation | 0.988 |
| SmallAreaHighGreyLevelEmphasis | -0.827 | SurfaceVolumeRatio | -0.944 |
| Idmn | -0.771 | GrayLevelNonUniformity.2 | 0.903 |
| Maximum2DDiameterRow | -0.445 | Contrast | -0.865 |
| RunLengthNonUniformityNormalized | -0.050 | RunLengthNonUniformityNormalized | -0.853 |
|  |  | LongRunLowGreyLevelEmphasis | 0.794 |
|  |  | DifferenceVariance | -0.698 |
|  |  | RunVariance | -0.659 |
|  |  | Imc1 | 0.637 |
|  |  | SizeZoneNonUniformityNormalized | -0.616 |

Note. — Intercept $\theta_{0}$ is 0.0.294 in the TBR_5-15_ model, and 0.607 in TBR_20-40_ model.

**Supplementary Figure**

**
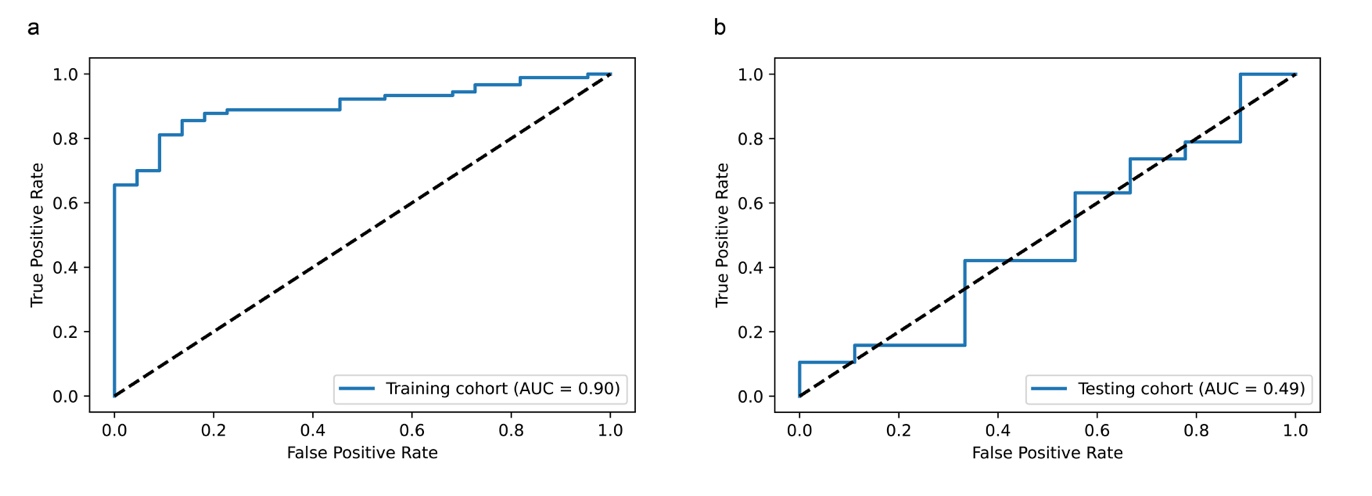
**

**Figure S1.** (a) TBR_20-40_ model reached an AUC of 0.90 in the training cohort, and (b) an AUC of 0.49 in the testing cohort. AUC, area under the receiver operating characteristic curve.
